# Commentary on “Endovascular Therapy for Stroke Due to Basilar-Artery Occlusion”

**DOI:** 10.1007/s00270-021-02917-y

**Published:** 2021-07-20

**Authors:** Kornelia Kreiser

**Affiliations:** Departement of Radiology/Neuroradiology, RKU - Universitäts- und Rehabilitationskliniken Ulm gGmbH, Oberer Eselsberg 45, 89081 Ulm, Germany

The superiority of endovascular thrombectomy (EVT) over best medical treatment (BMT) for large vessel occlusions (LVO) in the anterior circulation has been proven by a total of 5 studies since 2015 [1]. To increase the level of evidence also for LVOs of the posterior circulation, a number of efforts have been initiated in recent years with varying degrees of success.

One trial was stopped early due to poor recruitment rates [2]. A large registry from China was able to show a considerable advantage of EVT over BMT, but did not reach evidence level I due to the lack of randomization [3].

Now, the eagerly awaited results of the BASICS trial were published but seem to leave more questions unanswered than answered. The authors, mainly Dutch, could not find any superiority of EVT after completion of the study with 300 treated patients. Instead of the expected difference of 16%, there was only a 6.5% advantage in EVT patients who achieved a favourable outcome, i.e. an mRS of ≤ 3.

But if you look in detail, it is not so much the rate of EVT patients with favourable outcome that deviates from expectations (44.2% instead of 46%). Rather surprising is the unexpectedly favourable performance of BMT patients with 37.7% instead of expected 30%. The rate of symptomatic intracranial haemorrhage was also comparable to study results for the anterior circulation with 4.5% in the EVT group, but unexpectedly low in the BMT group with only 0.7%, even if this difference did not meet statistical significance.

Some limitations of the study are already listed by the authors. First, there is the long period of patient recruitment of just over 8 years. In addition, 29.2% of patients who actually met the inclusion criteria were ultimately treated outside the study and 79% of them received EVT. Unfortunately, there is no information on the reasons, but it is likely that personal preferences of relatives, patients, and the interventionalist himself have played a major role since 2015.

For ethical reasons, it would be understandable that especially young patients should be protected from randomisation because they did not want to be denied the chance of the most aggressive therapy. Perhaps, rather mild affected persons with smaller thrombus masses were included at all. Here, a higher success rate for BMT can certainly be expected than in those with long occlusions [4]. However, this deprives EVT of the chance to prove itself in a comparison. A closer look at the patients who were not included is absolutely necessary.

Further, the data are not very productive with regard to subgroup analyses, which the authors themselves restrictively note. In particular, the question of the underlying aetiologies and the need for stent implantation are only dealt with descriptively, although this might be the key to understanding the results.

The diagnosis "basilar artery thrombosis" is composed of at least two very different aetiologies: "embolism" and "atherosclerosis". The interventional procedure, but even more so the prognosis, can vary considerably. Due to the small proportion of only 10% of all ischaemic strokes, it is therefore even more difficult to separate the respective subgroups clearly and to achieve a high level of evidence for each of them.

We will see if the confusion subsides or increases after completion of other still running studies. One ongoing trial in China [5] only includes patients more than 6 h after symptom onset, even if i.v. thrombolysis has already been administered unsuccessfully. This probably excludes mainly those patients with uncomplicated occlusions, resolving under BMT and favours those with underlying stenoses. If so, EVT may have an advantage over BMT, at least in this population, but the outcome of all patients will be inherently worse and the differences may not be sufficiently detectable.

In conclusion, the question remains whether the highest level of evidence can be obtained exclusively through randomisation? Perhaps large registries are able to reflect reality much more reliably.
